# Cerebrospinal fluid metabolomics in autistic regression reveals dysregulation of sphingolipids and decreased β-hydroxybutyrate

**DOI:** 10.1016/j.ebiom.2025.105664

**Published:** 2025-03-25

**Authors:** Jingya Yan, Velda X. Han, Hannah F. Jones, Timothy A. Couttas, Beverly Jieu, F. Markus Leweke, Jennifer Lee, Catherine Loi, Richard Webster, Kavitha Kothur, Manoj P. Menezes, Jayne Antony, Tejaswi Kandula, Michael Cardamone, Shrujna Patel, Sushil Bandodkar, Russell C. Dale

**Affiliations:** a Kids Neuroscience Centre, The Children’s Hospital at Westmead, Faculty of Medicine and Health, University of Sydney, NSW, Australia; b School of Mathematical and Physical Sciences, Faculty of Science, University of Technology Sydney, NSW, Australia; c Clinical School, The Children’s Hospital at Westmead, Faculty of Medicine and Health, University of Sydney, NSW, Australia; d Khoo Teck Puat-National University Children’s Medical Institute, National University Health System, Singapore, Singapore; e Department of Paediatrics, Yong Loo Lin School of Medicine, National University of Singapore, Singapore; f Starship Hospital, Centre for Brain Research, Faculty of Medical and Health Sciences, University of Auckland, New Zealand; g Neuroscience Research Australia, Randwick, NSW, Australia; h Brain and Mind Centre, The University of Sydney, NSW, Australia; i Department of Endocrinology, The Children’s Hospital at Westmead, NSW, Australia; j TY Nelson Department of Neurology and Neurosurgery, The Children’s Hospital at Westmead, The University of Sydney, Westmead, New South Wales, Australia; k Department of Neurology, Sydney Children’s Hospital Network, Sydney, NSW, Australia; l Department of Biochemistry, The Children’s Hospital at Westmead, NSW, Australia

**Keywords:** Neurodevelopmental disorders, Metabolomics, Lipidomics, Inflammation

## Abstract

**Background:**

Autism is highly heritable, however actionable genetic findings are only found in a minority of patients. Many people with autism suffer loss of neurodevelopmental skills, known as autistic regression. The cause of regression is poorly understood, and the diagnostic and therapeutic pathways are lacking.

**Methods:**

We used untargeted metabolomics using a UPLC-Q-Exactive-HFx Mass Spectrometry to examine cerebrospinal fluid (CSF) from twenty-two patients with autistic regression compared to sixteen controls with neurodevelopmental disorders (but not autistic regression) and thirty-four controls with other neurological disease (headache, encephalitis, epilepsy). The twenty-two patients with autistic regression consisted of two groups: early (infantile) autistic regression <2 years of age (n = 8), and later regression of skills >4 years of age, often in the context of pre-existing developmental concerns (n = 14). Metabolites of interest were then quantified and validated using targeted assays.

**Findings:**

Untargeted case-control studies revealed good separation of patients from controls using multivariate analysis. β-hydroxybutyrate was significantly decreased in the CSF of patients with autistic regression, and the findings were validated using a targeted β-hydroxybutyrate assay. The sphingolipid, sphingosine-1-phosphate was significantly elevated in the discovery case-control studies, and sphingolipid metabolism pathways were also significantly dysregulated. We therefore developed a targeted metabolite assay of forty sphingolipids. After FDR correction, 21 of the 40 sphingolipids were significantly dysregulated (p_FDR_ < 0.05) (*Benjamini-Hochberg correction*) in autistic regression compared to the neurodevelopmental controls, and 26 of the 40 sphingolipids were significantly dysregulated in autistic regression compared to other neurological controls, with elevated ceramides, hexosylceramides, sphingosines (including sphingosine-1-phosphate), and sulfatides. By contrast, sphingomyelin levels were generally decreased in autistic regression.

**Interpretation:**

Our data shows the potential utility of CSF metabolomics in the context of autistic regression, a clinical syndrome which has historically lacked pathophysiological biomarkers and disease modifying therapies.

**Funding:**

Financial support for the study was granted by Dale NHMRC Investigator grant APP1193648, 10.13039/501100002337Petre Foundation, 10.13039/501100022099Cerebral Palsy Alliance, and Ainsworth and SCHF Neuroscience grant scheme.

**Research in context**

**Evidence before this study**

Within the landscape of neurodevelopmental disorders, autistic regression affects about 30% of children, however, the outcomes are detrimental, often leading to long-term neurodisability. Metabolomics has emerged as a promising tool in autism spectrum disorders to explore the pathophysiological mechanisms and reflect the interactions between genetic and environmental influences. Given the heterogeneity and neurologic manifestations of autistic regression, this represents an important area of research. This study presents an investigation of the CSF metabolomic profile in autistic regression.

**Added value of this study**

In this study, we show alterations in CSF β-hydroxybutyrate and the dysregulation of sphingolipid metabolism pathways in autistic regression. Sphingosine-1-phosphate and β-hydroxybutyrate were key metabolic changes. They could represent pathophysiological biomarkers of autistic regression and potential therapeutic targets in the future.

**Implications of all the available evidence**

There are very few studies comparing alterations in CSF metabolite profiles in autism spectrum disorders and the literature in autistic regression is even more scarce. The reproducibility of CSF metabolomics findings in our discovery and targeted studies provides insight into the pathophysiology of autistic regression, with the potential for clinical and therapeutic translation.

## Introduction

Autism is a highly heritable disease and is increasing in prevalence partly due to increased diagnosis and recognition, and partly due to unknown factors.^1^ However, rare, highly penetrant gene variants are found in only <10–35% of studied autism cohorts, and this variable diagnostic yield is influenced by genomic methodology and the presence of comorbidities such as intellectual disability or dysmorphic features.^2^ Rather than highly penetrant gene variants, many patients are presumed to harbour common ‘vulnerability’ genetic variants,^3^ and these common genetic variants are shared with many common developmental disorders^4^^,^^5^ and mental health^6^^,^^7^ disorders such as schizophrenia, ADHD, bipolar disorder and depression. Therefore, in clinical practise, investigation often fails to yield an ‘actionable’ genetic finding in autistic spectrum disorder, unless there are co-existent syndromic or dysmorphic features, or intellectual disability. It is proposed that gene–environment interactions are also an important yet under-investigated disease mechanism in autistic spectrum disorder.^8^^,^^9^

Loss of developmental skills in the context of autism is commonly described by caregivers, and autistic regression is the best described and accepted terminology, although standardised definitions do not yet exist.^10^ Indeed, previous studies have shown that the most valuable diagnostic tool to support a diagnosis of autistic regression is parental videos before and after the regression.^11^^,^^12^ Autistic regression describes the loss of previously acquired neurodevelopmental skills (language and/or social), and most commonly occurs in the second year of life.^10^^,^^13^ Children with autistic regression can either have normal preceding development, or preceding developmental concerns. The hallmark of autistic regression is the loss of skills such as social interaction (change in eye contact or communication intent), language (loss of previously acquired language skills), or the appearance of new repetitive stereotypical behaviours or behavioural changes.^10^ However, loss of developmental skills in the context of autism can occur after the second year of life, and present with loss of social, language and cognitive skills, and sometimes acquisition of problems such as obsessive-compulsive disorder, tics or executive dysfunction. In a majority of children with autistic regression, initial investigation including MRI brain, lumbar puncture, genetic testing and routine tests are negative or normal, and there are unclear therapeutic pathways other than routine developmental support and treatment of co-morbid behaviours.^10^

Metabolomics has emerged as a promising tool to explore underlying biochemical disruptions and to discover novel biomarkers for neurodevelopmental disorders. We performed untargeted followed by targeted CSF metabolomics to discover biomarkers in children with autistic regression, with the aim to provide insights into disease mechanisms, and potential future therapeutic opportunities.

## Methods

### Study design and participants

#### Autistic regression cohort

##### Inclusion criteria

All children with autistic regression (n = 22) had one or more episodes of losing previously established skills; they lost spoken language (such as a child who was regularly speaking phrases who stops speaking) and/or lost social and communication abilities (such as a child previously using gestures and making regular eye contact who loses such social responsiveness).^10^ This is a retrospective study, with participants recruited across the Sydney Children’s Hospitals Network (SCHN) from the Children’s Hospital at Westmead (CHW) and Sydney Children’s Hospital Randwick between 2018 and 2023. The SCHN is a tertiary neurology referral centre in NSW, Australia, a state of 8 million people. The CSF neurochemistry laboratory at CHW receives approximately 500 samples annually and is a state and national CSF referral centre. Residual CSF left after routine testing is stored at −80 °C and available for research, subject to ethics requirements. All patients were assessed by paediatricians and fulfilled the DSM-5 criteria for autistic spectrum disorder and were assessed with Autism Diagnostic Observation Schedule (ADOS), to fulfil the autistic spectrum disorder criteria according to the National Disability insurance scheme in Australia. All patients and controls underwent CSF examination after a 4-h fasting period, as the procedure required general anaesthesia or sedation for all individuals.

The patients (n = 22) underwent neurological investigations including neuroimaging, genetic testing, and cerebrospinal fluid examination to exclude neurodegenerative conditions such as leukodystrophy, or acute or chronic encephalitis. These investigations were directed by the treating clinicians (Tables 1 and 2), and all twenty-two children had MRI brain which provided diagnostic information in no patients; nineteen patients were reported normal, and three patients had findings of no clear diagnostic significance (non-specific white matter hyperintensity, Rathke’s cyst, arachnoid cyst, all n = 1). Genetic testing was driven by the clinician based on clinical findings, rather than a protocol; Only 3 of 22 patients had no genetic testing. Of the nineteen children who had genetic testing, ten had a CGH microarray, ten had an exome/genome sequencing (ID/ASD panel n = 2, singleton exome n = 3, trio exome n = 4, trio genome n = 1), and five patients had specific gene testing (Rett syndrome n = 2, Fragile X n = 3). The genetic testing in these patients (n = 19) revealed no actionable findings which was deemed pathogenic or likely pathogenic.

**Table 1:** The number, sex distribution, age (median and range) and specific aetiology of autistic regression and control groups in each cohort study.

	Cohort	Number	Males: females	Age mean, median, range (years)	Aetiology
Untargeted metabolomics	Autistic regression cohort 1	11	11 M	8.1, 7.6 (3.4–15.4)	Table 2
	Control cohort 1	11	9 M:2 F	8.2, 8.6 (0.5–16.2)	Headache (intracranial hypertension, migraine, n = 4), neurogenetic (n = 2), neurodevelopmental (n = 2), epilepsy, visual disturbance, cerebral palsy (all n = 1)
	Autistic regression cohort 2	11	8 M:3 F	7.9, 7.5 (1.5–14.4)	Table 2
	Control cohort 2	11	7 M:4 F	9.3, 10.3 (0.5–14.5)	Headache (intracranial hypertension, migraine, n = 5), neurogenetic (n = 3), neurodevelopmental (n = 3)
Targeted sphingolipid	Autistic regression	14	12 M:2 F	7.3, 7 (range 1.5–13)	Table 2
	Neurodevelopmental controls	16	8 M:8 F	5.1, 2.9 (range 0.4–16.2)	Neurodevelopmental disorder (all global delay or intellectual disability, without autistic regression) (n = 16)
	Other neurological controls	34	13 M:21 F	8.0, 0.1 (range 0.1–16.5)	Epilepsy (genetic epilepsy, status epilepticus) (n = 10), neuroinflammation (encephalitis, multiple sclerosis) (n = 12), headache (intracranial hypertension, migraine) (n = 12)

**Table 2:** Clinical data of autistic regression patients n = 22.

Case, sex	FH NDD	FH other	Pregnancy	Pre-regression development	Regression age (yr)	Regression trigger	Regression phenotype	Further regression episodes	Length follow-up (post regression)	Current diagnoses
1:1, M	–	–	–	No words, normal socialisation and development	1.5	–	Loss eye contact, stopped playing, lost interaction	3 y (URTI and skin infection association): onset anxiety, SIB	8.8 y	ASD, ID, SIB
1:2, M	ASD, ADHD (sister)	Depression, asthma (mother)	Pre-eclampsia	normal	1.15	Fever, mouth ulcers, rash	Loss social interaction, repetitive behaviour, irritability	Multiple per year: cognition, attention, OCD, tics	9.8 y	ASD, ADHD, Tourette, OCD, anxiety
1:3, M	–	–	–	normal	1.5	Pneumonia (hospitalised)	Lost single words and simple phrases, loss social interaction, play became restricted	Further setbacks and loss of language (no trigger)	3.5 y	ASD, anxiety, sensory
1:4, M	ASD (mother, sister)	Recurrent encephalitis (father)	–	ASD, mild ID	5.5	URTI	Loss of language, OCD, talking gibberish, agitation, repetitive behaviour and tics	–	4 y	ASD, ID, OCD, tics, separation anxiety, SIB
1:5, M	–	Hashimoto thyroiditis (mother)	New onset hypothyroidism, gestational diabetes	ASD	6.4	Fever, rash	Loss of language (sentences to phrase), loss memory/learning, anxiety, aggression, ritualistic behaviour	–	7.9 y	ASD, ID, OCD, tics, ADHD
1:6, M	ASD (2 sisters)	–	Gestational diabetes, pre-eclampsia, hyper-emesis	ASD (high functioning)	7	–	Cognitive and language decline, OCD, anxiety, sensory and auditory processing issues, encopresis, enuresis	Ongoing fluctuations	7.1 y	ASD, ADHD, OCD, tics, anxiety, sensory
1:7, M	ADHD (brother)	–	–	ASD	7	–	Cognitive and memory decline, loss of speech, concentration and hyperactivity, and complex stereotypies	–	10 y	ASD, ID, ADHD
1:8, M	–	–	–	ASD	9	–	Inattention, language loss, tics, OCD, anxiety, sensory	–	5.5 y	ASD, ID, Tourette, OCD, anxiety, sensory
1:9, M	–	Domestic abuse, PTSD (mother)	–	ASD	10	–	Speech, cognition, motor decline	–	4 y	ASD, ID, motor disability, neuropathy
1:10, M	ASD (brother)	–	–	ASD, epilepsy	8	TBI	Speech loss, anxiety, self-injury, repetitive behaviour	Ongoing fluctuations	3.5 y	ASD, ID, epilepsy, SIB, aggression
1:11, M	ADHD (sister, mother), OCD, PTSD (mother)	Coeliac disease (mother)	Hyperemesis	normal	1.4	4 infections in 6 weeks (ear, throat, HFMD, rash)	loss of play, loss understanding name, body part, stopped responding to name, agitated, poor sleep,	Further regression age 3 y (rash, loss speech, reduced eye contact, OCD, repetitive behaviour)	3.8 y	ASD, OCD, tics, anxiety, SIB
2:1, M	–	Anxiety (mother, father, brother)	–	ASD level 1	11	TBI then Covid19 infection	Loss of speech (mutism), sleep change, anxiety, inattention, SIB	–	3.4 y	ASD, anxiety, depression
2:2, M	–	–	Gestational diabetes, pre-eclampsia	ASD, mild ID	6	impetigo	Cognitive and language decline, inattention, OCD, tics, anxiety	Ongoing fluctuations with skin infections	2.5 y	ASD, mild ID, ADHD, tics, OCD
2:3, M	ASD (sister, brother), ADHD (sister, brother), PTSD (father)	CVID (mother, sister, proband), systemic lupus erythematosus (mother)	–	ASD, ADHD, epilepsy	8	–	Cognitive and language decline, memory loss, seizures, agitation	Further regression age 13 y: language loss, motor decline (wheelchair)	8.1 y	ASD, ID, motor disability, anxiety, OCD, CVID (on IVIG)
2:4, F	ASD (paternal cousin)	–	–	normal	1.25	Unexplained 5 week vomiting illness	Loss eye contact, loss socialisation, repetitive behaviour started, irritable	–	1 y	ASD, OCD, repetitive behaviours
2:5, M	–	–	–	normal	1.5	–	Loss socialisation, imaginative play, cognitive plateauing, challenging behaviour	–	2 y	ASD, ID, challenging behaviour
2:6, M	Epilepsy (mother, brother)	–	–	Mod DD, ASD level 2	6	Influenza vaccine	Loss language, less social, more isolated, more inattentive, more stereotypy, sleep change, urine incontinence	–	5 y	ASD, ID, aggression, occ seizure,
2:7, F	Schizophrenia (father)	Immune NK disorder (mother)	–	Normal, mild separation anxiety	4.5	3 infections in 3 weeks	Loss socialisation, confusion, hallucinations, eating restriction, incontinence, anxiety	Further infection associated regressions (agitation, aggression, enuresis, encoporesis, some seizures)	5.5 y	ASD, ID (mild), OCD, ADHD, separation anxiety
2:8, M	–	–	–	Global DD	7	–	Loss language, restricted play, sleep, incontinence (urine, bowel), aggression and irritable	–	3 y	ASD, ID (mod), ADHD, challenging behaviour
2:9, M	ASD (brother, sister), tics-OCD (father)	Alopecia (father), coeliac (mother)	–	normal	1	2 infections (chickenpox, croup) and 2 vaccine (meningococcal, MMR)	Loss eye contact, loss language, repetitive behaviour	Abrupt onset OCD age 13 y	15.2 y	ASD, ID, OCD, SIB
2:10, M	–	Hashimoto thyroiditis (proband)	–	normal	0.9	Prolonged fever and vomiting illness	Loss eye contact, loss interaction, loss vocalisation	–	10 y	ASD, ID, OCD, tics
2:11, M	–	–	–	Normal (borderline language)	5	Fever and vomiting illness nos	Loss eye contact, own world, jargon speech, loss language, severe hyperactivity, hallucinations	–	3.3 y	ASD, ID (mild), tics, OCD, ADHD

ADHD: attention deficit hyperactivity disorder, ASD: autistic spectrum disorder, CVID: combined variable immune deficiency: family history, ID: intellectual disability, NDD: neurodevelopmental disorder, nos: not otherwise specified, OCD: obsessive-compulsive disorder, SIB: self-injurious behaviour, URTI: upper respiratory tract infection.

CSF testing was performed median two years after onset of first regression episode (mean 2.7 years, range 0.2–13 years). Routine diagnostic CSF testing was normal: there was no CSF pleocytosis in all 19/22 tested (median 0, mean 0.8, range 0–4 cells/mm3). CSF neopterin, an acute phase neuroinflammatory biomarker, was not elevated in all 19/22 with data available (median 18, mean 18, range 7.4–28, normal < 30 nmol/l).

#### Controls

Inclusion criteria: We recruited controls for the untargeted metabolomics and targeted lipidomics, the controls had a CSF sample to investigate their neurological disorder that did not include autism (including on follow-up) (Table 1). The controls were not significantly different to the patients with autistic regression in age, sex distribution and weight/BMI. For presentation, the control group was separated into 2 subgroups.

1. ‘Neurodevelopmental disorders’ (n = 16) consisted of children with neurodevelopmental disorders affecting motor, cognitive, or language development, but without autistic regression. Many of these cases had a defined genetic cause for their neurodevelopmental disorder.
2. ‘Other neurological disorders’ (n = 34) were presented together and consisted of three major subgroups:
  a. Headache subgroup (n = 12) consisted of children diagnosed with idiopathic intracranial hypertension or migraine. This is the closest subgroup to ‘healthy controls’, as these children require a CSF and generally have no other neurological disorders.
  b. Inflammatory subgroup (n = 12) consisted of children with encephalitis and multiple sclerosis and was selected based on our hypothesis that inflammation may play a role in autistic regression.
  c. Epilepsy subgroup (n = 10) is a common clinical problem that requires CSF examination and consisted of children with genetic epilepsy or status epilepticus.

### Chemical and reagents

For the untargeted metabolomics, an internal standard mixture consisted of D_3_-tryptophan, D_4_-kynurenine, 13C6-arginine was supplied by Toronto Research Chemicals (Toronto, Canada).

For the targeted lipidomics, a mixed internal standard consisted of d17:1 Sphingosine, d17:1 Sphingosine-1-Phosphate, d18:1/17:0 Sphingomyelin, d18:1/17:0 Ceramide, and was purchased from Sapphire Bioscience (Sydney, Australia).

HPLC grade acetonitrile and methanol, ammonium formate and methyl tert-butyl ether were purchased from Sigma Aldrich (Sydney, Australia). Fresh Type I Water from a Millipore Elix Essential and Synergy-UV were used. Formic acid was supplied by Fisher Chemical (Fair Lawn, New Jersey). The catalogue numbers of the reagents and chemicals are provided in Supplemental Table S1.

### Untargeted metabolomics

We performed untargeted metabolomics on a first cohort of autistic regression cases versus controls (n = 11 v n = 11), and then a second independent case-control cohort study (n = 11 v n = 11) (Table 1). The study was conducted using previously stored CSF samples that have not been used in any previous studies. The collection of CSF samples was conducted using an aseptic technique and frozen within 1 h of sampling and stored at −80 °C. The CSF tube analysed for this study was not used for routine testing and was not previously thawed before the untargeted metabolomic studies.

The sample preparation and metabolomic profiling were performed in accordance with the previously reported method of Yan et al.^14^ Briefly, 100 μL of human CSF was deproteinised with methanol, followed by mixing, sonication and centrifugation in Eppendorf tubes. The collected supernatant was evaporated to dryness under nitrogen and reconstituted for analysis using a Thermo Scientific Vanquish system coupled to a Q Exactive HF-X Hybrid Quadrupole Orbitrap Mass Spectrometer (Thermo Fisher Scientific, San Jose, CA, USA). The chromatographic separation of metabolites was achieved using the Acquity UPLC HSS T3 Column (2.1 mm × 150 mm 1.7 μm particle size) and a flow rate of 0.30 mL min^−1^. The mobile phases consisted of 20 mM ammonium formate in water (A) and 20 mM ammonium formate and 0.01% formic acid in acetonitrile (B). The gradient programme was: 0–1 min (2% B), 1–4.5 min (2−20% B), 4.5–10 min (20–40% B), 10–15 min (40−99% B), 15–16 min (99-2% B), and 16–20 min (2% B). The mass spectrometer was acquired in the all-ion fragmentation mode using positive electrospray ionisation under a high mass resolution of 120 000 and a detection scan range from m/z 50 to 800. In both cohort studies, four quality control samples were injected and analysed. Quality control samples for each case-control study were prepared from a pooled mixture of equal volumes of CSF samples and inserted randomly across the run. We applied the following batch effect removal methods; (i) internal standard normalization to ensure that measurements are comparable across the two case-control studies and (ii) the use of QC data to account for signal drift and batch-to batch variations.^15^

Metabolite identification and analysis was conducted using Compound Discoverer 3.3 software (Thermo Fisher Scientific Inc.). Automated peak detection, alignment, retention time correction, normalisation, identification, and determination of differences between raw data sample sets were carried out to curate the large quantity of data. To reduce false positive and negative features in our data processing step, the eliminated mass spectral features were set to consider features present in at least 75% of the samples.^16^ The databases used for metabolite identification were the Human Metabolome Database, mzCloud, ChemSpider, and self-built in-house database for qualitative analysis. The pre-processed data sets were extracted from the Compound Discoverer and were normalized against internal standards. Subsequently, input for multivariate and univariate analysis we used Metaboanalyst 5.0.^17^ The multivariate analyses consisted of partial least squares discriminant analysis (PLS-DA), fold change and heatmap. Significant metabolites statistically driving the separation between the groups were obtained through analysis of variance (ANOVA) and Fisher’s least significant difference (LSD) post hoc analysis at a p-value cut-off of 0.01 and false discovery rate at 5% with *p*_FDR_ values < 0.05 as statistically significant. These thresholds are commonly used in metabolomics studies.^18^^,^^19^

### Targeted metabolomics of sphingolipid metabolism

Sphingolipids were extracted from human CSF samples and analysed by the method as described by Wong et al.^20^ Briefly, sphingolipid quantification for forty compounds across sphingosine, sphingosine-1-phosphate (S1P), ceramides, hexosylceramides, sphingomyelins and sulfatides was performed by selected reaction monitoring using the Thermo Scientific Vanquish system coupled to a TSQ Altis triple quadrupole mass spectrometer (Thermo Fisher Scientific, San Jose, CA, USA). Lipids were separated using the Agilent Eclipse Plus C8 column (2.1 mm × 100 mm 1.8 μm particle size) and a 25 min gradient program. The lipids were detected on the mass spectrometer in the selected reaction monitoring mode using positive electrospray ionisation. Xcalibur was used for peak integration and peaks were normalised as ratios to their class-specific internal standard.

### β-Hydroxybutyrate assay

One of the metabolites in the untargeted study was β-hydroxybutyrate, which was measured as a targeted metabolite by the single analyte LiquiColor assay (EKF Diagnostics—Stanbio ©, Texas U.S.A.) and analysed on the Infinite 200 PRO NanoQuant Plate (Tecan, Switzerland) set at 505 nm. Briefly, 90 μL of CSF was mixed with 700 μL of reagent, followed by 125 μL of a catalyst reagent in polystyrene multi-dishes (Thermo Fisher Scientific, Australia). The dish was incubated for 10 min at 25 °C and measured in the microplate reader. As adequate CSF was not available in all samples, β-hydroxybutyrate was quantified in patients with autistic regression (n = 17 of 22 with CSF available from cohort 1 and 2) and controls (n = 12 of 22 with CSF available from controls cohorts 1 and 2).

### Statistics

Statistical analyses were performed using GraphPad Prism 8 (GraphPad, San Diego, USA) and SPSS version 26 (IBM Corp. Armonk, NY, USA). As the data was not normally distributed, non-parametric statistics (Mann–Whitney U test) were performed. GraphPad Prism was used for analyses of putative marker concentrations which, due to their substantially skewed distributions, are presented on the log2 scale. Due to the multiple comparisons in the targeted lipidomics study of sphingolipid metabolism, we used a false discovery rate approach with a cut-off of 0.05 (*Benjamini-Hochberg correction*) for the pairwise comparisons.

### Ethics

The Sydney Children’s Hospitals Network Ethics Committee approved this study (LNR/14/SCHN/275; 2019/ETH06182). Families were contacted and provided written informed consent to use the residual CSF still available after routine testing, as per ethics protocol.

### Role of funders

Funding sources had no role in the design of this study, and did not have any role during its execution, analyses, interpretation of the data, or decision to submit results.

## Results

### Autistic regression cohort

Of twenty-two patients, 20/22 were male, 11/22 had a positive family history of significant neurodevelopmental disorders in first degree family members (7/22 had first degree FH of autism) and 5/22 had a first-degree family history of mental health disorders. In addition, 7/22 had a first-degree family history of autoimmune conditions, and 5/22 had notable complications of pregnancy (Table 2). The mean age of regression was 5 years, median 5.5 years (range 0.9–11 years), and there were two main groups; those who regressed in infancy (<2 years) who had normal or near normal preceding development (n = 8), and then a second older group (>4 years) who typically had some neurodevelopmental concerns (including autism) who then had a regression of socialisation, language, cognition, attention, repetitive behaviour, sometimes with associated new onset OCD or tics (n = 14). 14/22 had an associated, apparently triggering event in the weeks before the change in neurodevelopment: a majority of the trigger events were febrile illnesses, although four patients had multiple apparent triggers in a short period (e.g., traumatic brain injury and COVID19 infection, multiple infections, or multiple infections plus vaccinations).

All patients had loss of social interaction or loss of purposeful language in the context of preceding normal development, or in the context of pre-existing autism (as per inclusion criteria). The predominant features of the regressive episode was loss or alteration of language (n = 15), altered social interaction (n = 11), new irritability/aggression/SIB (n = 11), cognitive or memory decline (n = 9), new anxiety/OCD (n = 8), new repetitive or ritualistic behaviour (n = 7), new hyperactivity/inattention (n = 6), change in play (n = 5), sleep change (n = 4), new incontinence (n = 4), tics (n = 3), hallucinations (n = 2), sensory issues (n = 2), and motor decline, eating restriction and seizures (each n = 1). 10/22 patients had more than one regressive episode (defined as further loss of previously acquired language or social developmental skills), and six of the patients had multiple regressive episodes with fluctuations in neurodevelopment.

After a mean follow-up of 5.6 years after first regression (range 1–15.2 years), all 22/22 maintained an autism diagnosis, 16/22 had intellectual disability, 13/22 OCD, 9/22 tics or Tourette syndrome, 7/22 ADHD and 5/22 self-injurious behaviour.

### Untargeted metabolomics

**Fig. 1:**
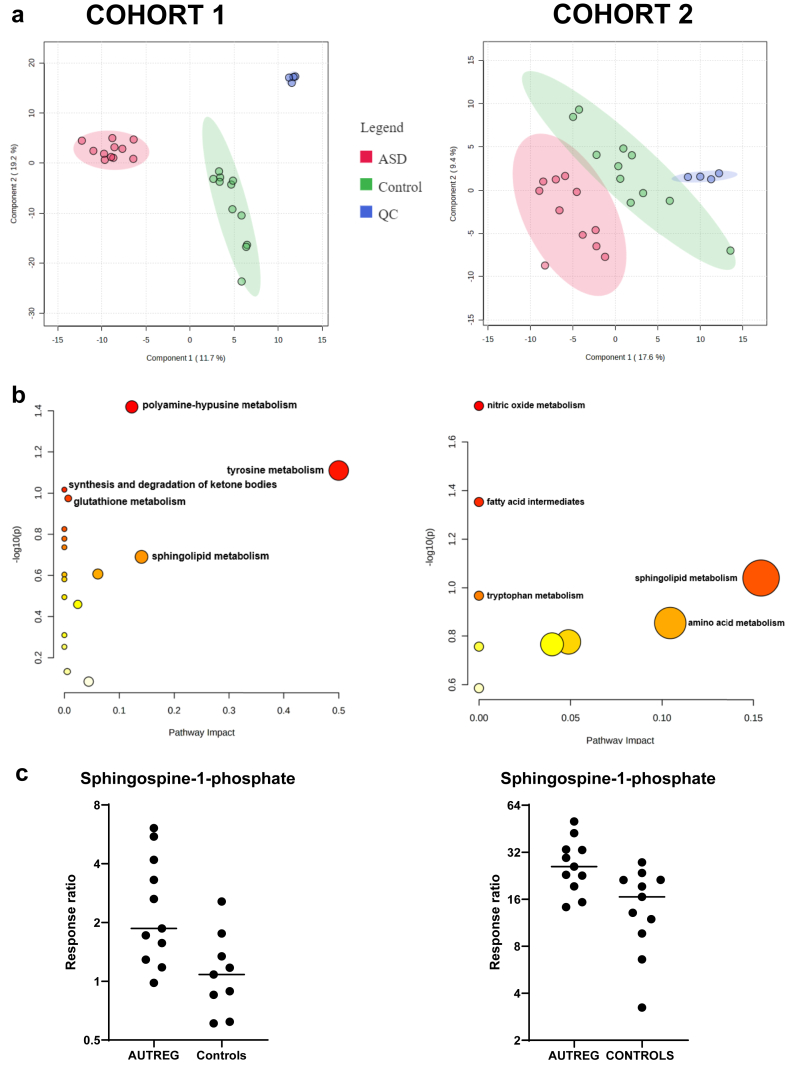
Summary of statistical analyses of cerebrospinal fluid cohort studies, cohort 1 (left) and cohort 2 (right) using ultra-performance liquid chromatography coupled to high resolution mass spectrometry. (a) Partial least squares discriminant analysis score plots of patients with autistic regression (red dots), control patients (green dots) and quality control samples (blue dots) showing clustering of the respective groups. (b) Pathway analysis of statistically significant metabolites after FDR correction and a power analysis: sphingolipid metabolism is significantly dysregulated in both cohort 1 and 2. (c) Elevation of sphingosine-1-phosphate in the autistic regression group compared to controls showing p = 0.0097 (cohort 1; AUTREG, n = 11; Controls, n = 11) and p = 0.0083 (cohort 2; AUTREG, n = 11; Controls, n = 11) (ANOVA and Fisher’s LSD FDR-adjusted). The response ratio is the ratio of *peak area of metabolite: internal standard peak area*.

We performed two case-control untargeted studies-the first was a ‘discovery case-control study’ consisting of eleven patients and eleven controls, and the second was a ‘validation case-control study’ consisting of eleven further patients and eleven further controls, to see if we could reproduce findings. The PLS-DA data in both case-control studies 1 and 2 presented a tight cluster which indicated good reproducibility and ensured the robustness of the metabolomics analysis method (Fig. 1a).

In the untargeted case-control 1 study, metabolic differences were identified in 127 CSF metabolite levels (113 upregulated and 14 downregulated) between autistic regression and controls. ANOVA and Fisher’s LSD post hoc analysis revealed forty-four metabolites that were significantly involved in driving the discrimination between patients with autistic regression and controls. Following FDR correction (Table 3) we identified statistically significant metabolites from diverse metabolic pathways including sphingolipid metabolism, polyamine-hypusine metabolism, ketone bodies, tyrosine metabolism and glutathione metabolism (Fig. 1b). The levels of nine metabolites (p_FDR_ < 0.05) were elevated in the CSF of patients with autistic regression, and six metabolites were reduced (Table 3). In the autistic regression cases we also observed 12 statistically elevated drug metabolites (p_FDR_ < 0.05), as shown in Supplemental Table S2.

**Table 3:** Cohort 1 summary of ANOVA and Fisher’s least significant difference post hoc analysis at a p-value cut-off of 0.01, fold change and false discovery rate at 5% with *p*_FDR_ adjusted values < 0.05 as statistically significant for children with autistic regression (n = 11) compared to controls (n = 11).

Case-control study 1 metabolite	Change	Fold change	p_FDR_
Tyrosine	**↑**	3.94	0.04
Hypusine	**↑**	3.84	0.002
5-Methoxytryptamine	**↑**	3.44	0.002
Ornithine	**↑**	3.41	0.008
5-Oxoproline	**↑**	3.19	0.002
**Sphingosine-1-phosphate**	**↑**	**2.96**	**0.002**
3-Oxotetradecanoic acid	**↑**	2.63	0.03
3-oxolauric acid	**↑**	1.87	0.002
Valine	**↑**	1.60	0.006
**3-hydroxybutyrate**	**↓**	**0.21**	**0.006**
Arginine	**↓**	0.25	0.04
Creatine	**↓**	0.33	0.02
Adenine	**↓**	0.41	0.01
Homovanillic acid	**↓**	0.37	0.002
**Sphinganine**	**↓**	**0.44**	**0.006**

↑ represents elevation and ↓ represents decreased levels. The changes in statistically significant drug metabolites are presented in Supplemental Table S1.

Significantly differentiating metabolites of interest are highlighted in bold.

For untargeted case-control 2 study, differences were identified in 114 CSF metabolites (78 upregulated and 26 downregulated) between autistic regression and controls. There were thirty-nine metabolites revealed to drive the discrimination between patients with autistic regression and controls using ANOVA and Fisher’s LSD post hoc analysis. After FDR correction (Table 4), significant pathways involved sphingolipid metabolism, nitric oxide metabolism, amino acid metabolism, fatty acids and tryptophan metabolism (Fig. 1b). The levels of eight metabolites (p_FDR_ < 0.05) were elevated in the CSF of patients with autistic regression and seven metabolites were reduced (Table 4). In addition, there were eight statistically significant drug metabolites (p_FDR_ < 0.05, 7 elevated and 1 decreased) in the autistic regression group (Supplemental Table S2).

**Table 4:** Summary for cohort study 2 of ANOVA and Fisher’s least significant difference post hoc analysis at a p-value cut-off of 0.01, fold change and false discovery rate at 5% with *p*_FDR_ adjusted values < 0.05 as statistically significant for children with autistic regression (n = 11) compared to controls (n = 11).

Case-control study 2 metabolite	Change	Fold change	p_FDR_
Ornithine	**↑**	3.10	0.009
Dimethylarginine	**↑**	2.93	0.008
Methionine	**↑**	2.54	0.03
Acetylcarnitine	**↑**	2.50	0.01
**Sphingosine 1-phosphate**	**↑**	**2.48**	**0.008**
Succinyl-glutamic acid	**↑**	1.80	0.04
Uric Acid	**↑**	1.72	0.05
Tryptamine	**↑**	1.68	0.01
Leucine	**↓**	0.39	0.04
3-methoxy-4-hydroxyphenylglycol	**↓**	0.41	0.03
Lysine	**↓**	0.46	0.03
**Sphingosine**	**↓**	**0.52**	**0.05**
Undecenoic acid	**↓**	0.57	0.03
**Sphinganine**	**↓**	**0.61**	**0.01**
Carnitine	**↓**	0.64	0.03

↑ represents elevation and ↓ represents decreased levels. The changes in statistically significant drug metabolites are presented in Supplemental Table S1.

Significantly differentiating metabolites of interest are highlighted in bold.

The main findings of the untargeted case-control studies were significantly elevated S1P in both studies (Fig. 1c), decreased sphinganine in both studies and decreased β-hydroxybutyrate in study 1 (Table 3, Table 4). Due to the diversity of chemical and physical properties of our primary metabolite findings, we were unable to perform targeted validation in a single assay or platform. In addition, given the limited volumes of CSF samples, not all patient and control samples could undergo quantitative targeted analyses. First, we validated the β-hydroxybutyrate findings, then given the two sphingolipid metabolites found in both case-control studies using untargeted metabolomics, we developed a panel of forty sphingolipids.

### β-Hydroxybutyrate analysis

**Fig. 2:**
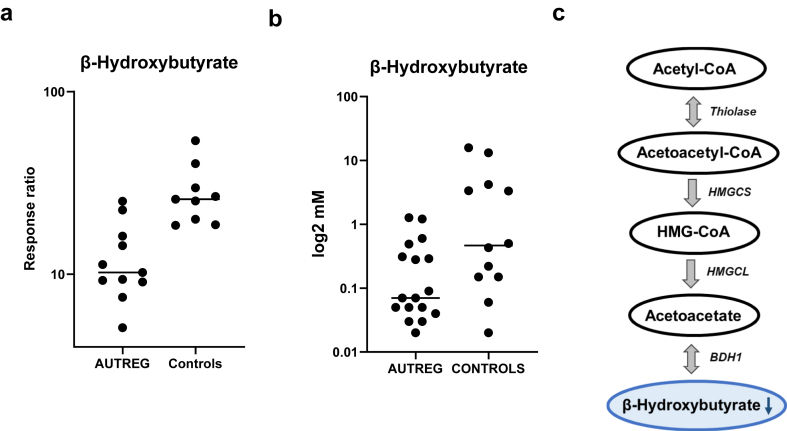
β-hydroxybutyrate data. (a) Untargeted metabolomics of patients with autistic regression (AUTREG, n = 11) are compared with controls (n = 11) showing β-hydroxybutyrate is reduced in the autistic regression group compared to controls (p = 0.0004) (ANOVA and Fisher’s LSD FDR-adjusted). The response ratio is the ratio of *peak area of metabolite: internal standard peak area*. (b) Validation of untargeted metabolomics study through quantification of β-hydroxybutyrate using a single analyte LiquiColor assay showing decreased β-hydroxybutyrate in patents with autistic regression (AUTREG, n = 17) and controls (n = 12) (p = 0.03) (Mann Whitney U test). (c) β-hydroxybutyrate pathway. HMGCS, 3-hydroxy-3-methylglutaryl-CoA synthase; HMGCL, 3-hydroxy-3-methylglutaryl-CoA lyase; BDH1, β-hydroxybutyrate dehydrogenase; HMG-CoA, 3-hydroxy-beta-methylglutaryl-CoA.

Residual CSF was available in seventeen autistic regression samples, and twelve control samples from the two case-control studies. The quantification of β-hydroxybutyrate confirmed statistically significant decreased concentrations in the autistic regression group compared to controls (p = 0.0347) (Mann Whitney U test) (Fig. 2).

### Sphingolipid metabolism analysis

**Fig. 3:**
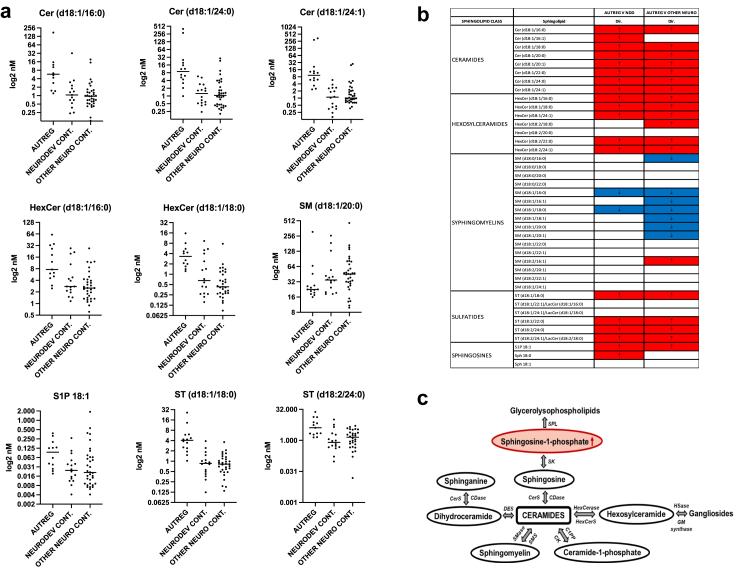
Targeted sphingolipids. (a) Targeted sphingolipids data of autistic regression group (AUTREG) compared to neurodevelopmental control group (NEURODEV CONT.) and ‘other neurological disorder’ control (OTHER NEURO CONT.) group (see Table 1). Representative statistically significant sphingolipids which are elevated in autistic regression are presented, including ceramides (Cer), hexosylceramides (HexCer), sulfatides (ST) and sphingosine 1 phosphate (S1P) (all < 0.05 according to *Benjamini-Hochberg correction FDR-adjusted*, see Supplemental Table S3 for values). By contrast, the sphingomyelin (SM) metabolites were generally decreased in autistic regression. (b) FDR adjusted p values of autistic regression (AUTREG) compared to other neurodevelopmental (NDD) controls and compared to ‘other neurological disorder controls are presented for all forty sphingolipids, with elevated values in red and decreased values in blue. Most sphingolipids were elevated in autistic regression, apart from sphingomyelin metabolites which were often decreased (full values in Supplemental Table S3) (c) Pathways of sphingolipid metabolism. SPL, SIP lyase; SK, sphingosine kinase; CDase, ceramidase; CerS, ceramide synthase; DES, dihydroceramide desaturase; HexCerase, hexosylceramidase; HexCerS, hexosylceramides synthase; HSase, hexosaminidase; CK, ceramide kinase; C1PP, ceramide-1-phosphate phosphatase; SMS, sphingomyelin synthase; SMase, sphingomyelinase.

Of the original twenty-two autistic regression samples, fourteen had residual CSF available for targeted sphingolipid analysis, and we used a neurodevelopmental disorder control group (n = 16) and an ‘other neurological disorder’ control group (n = 34) (total = 50 controls) (Table 1) for comparison with autistic regression. A panel of forty sphingolipids was categorised into eight ceramides, seven hexosylceramides, three sphingosines, six sulfatides and sixteen sphingomyelins. Comparing the autistic regression with the other neurodevelopmental disorder controls, twenty-one sphingolipids were statistically different after FDR *Benjamini-H. correction* (Fig. 3 and Supplemental Table S3). Comparing the autistic regression with the other neurological disorder control group, twenty-six sphingolipids were statistically different (Fig. 3 and Supplemental Table S3). In the sphingosine group, we confirmed elevation of S1P (Fig. 3). Representative sphingolipids are presented in Fig. 3a and the direction of change is presented in Fig. 3b. CSF S1P, ceramides, hexosylceramides and sulfatides were generally elevated in the autistic regression group compared to the two control groups. By contrast, the sphingomyelins were more likely to be decreased in the autistic regression group compared to the two control groups (Fig. 3b).

## Discussion

This study showed the utility of untargeted metabolomics as a discovery-driven approach to unravel metabolite signatures in complex and unexplained neurological disorders such as autistic regression. Autistic regression was selected as the cohort of interest due to the significant clinical challenges associated with diagnosis and investigation. The diagnostic yield of current routine investigations is poor, and many families are left with no explanation other than ‘regression happens in autism’. There is a poor understanding of why some children with autism regress, although there is some evidence to support mitochondrial or immune mechanisms.^21^^,^^22^ It is increasingly recognised that neurodevelopment is an active process dependent on brain connectivity and synaptic pruning.^23^^,^^24^ Synaptic pruning is an immunological process mediated by the immune cells of the brain, the microglia. Although there were clear ‘genetic’ vulnerabilities in the twenty-two patients with high rates of neurodevelopmental and neuropsychiatric disorders in family members, there were also high rates of immunological and environmental factors such as maternal autoimmunity, and the presence of pregnancy complications. Our hypothesis is that common neurodevelopmental disorders are often due to a combination of genetic and environmental factors that results in dysregulation of immune and brain cell function.

This study has pragmatic and ‘real life’ relevance, as children with autistic regression are highly likely to undergo a CSF examination due to diagnostic concerns surrounding the loss of developmental skills, compared to children with autism only (without regression). Our methodology constitutes a combination of untargeted and targeted metabolomic approaches. The former facilitated the discovery of molecular signatures that are hypotheses generating. The results from the untargeted approach served as a foundation for validation using targeted methods which are hypothesis driven and included subset analyses. Our primary findings are decreased β-hydroxybutyrate, and dysregulated sphingolipid metabolism.

There is a comprehensive metabolomics literature in autism,^25^, ^26^, ^27^, ^28^, ^29^, ^30^, ^31^ mainly in urine and plasma, with limited studies of autistic spectrum disorder (ASD) using human CSF.^32^^,^^33^ Common pathways reported to be dysregulated in ASD populations include amino acid metabolism (tyrosine, glutamate and nitric oxide pathways), energy metabolism (ketone bodies, fatty acids and Kreb cycle) and mitochondrial dysfunction. The heterogeneity in onset and progression of ASD across populations has limited the understanding of underlying mechanisms and consensus on biomarker results.^34^, ^35^, ^36^

The first main finding in our study was the presence of low CSF β-hydroxybutyrate detected in the untargeted metabolomics study, which was validated using a targeted assay. β-hydroxybutyrate is one of the most abundant ketone bodies formed from the metabolism of fatty acid oxidation, constituting 75% of the total circulating ketone bodies.^37^ The metabolism of β-hydroxybutyrate commences with the condensation of acetyl-CoA molecules to form acetoacetyl-CoA, catalysed by beta-ketothiolase. Subsequently, 3-hydroxy-3-methylglutaryl-CoA synthase (HMGCS) catalyses the condensation reaction to form 3-hydroxy-beta-methylglutaryl-CoA (HMG-CoA), which is degraded by HMG-CoA lyase (HMGCL) to produce acetoacetate. Acetoacetate is converted to β-hydroxybutyrate via β-hydroxybutyrate dehydrogenase (BDH1) (Fig. 2c). β-hydroxybutyrate is an important regulatory molecule that can be generated by most cells, but mainly by hepatocytes. Studies have shown β-hydroxybutyrate can modulate systemic and brain inflammation,^38^ however, its direct effects on microglia remain unclear.^39^ Ketones, and butyrate have demonstrated epigenetic and anti-inflammatory properties.^40^^,^^41^ β-hydroxybutyrate and butyrate are chemically similar and related short chain fatty acids.^42^ β-hydroxybutyrate is produced by the liver and butyrate by enteric butyric bacteria in the gastrointestinal tract. Similar to β-hydroxybutyrate, butyrate is a histone deacetylase (HDAC) inhibitor.^43^ Histone acetylation increases euchromatin which enables gene transcription, whereas histone deacetylation generally results in heterochromatin which inhibits gene transcription. Other than these epigenetic effects, butyrate is also known to have anti-inflammatory mechanisms, such as inhibitory effects on nuclear factor-κB (NF-κB) with secondary suppression of inflammatory gene expression.^44^ In addition, butyrate inhibits nod-like receptor pyrin domain 3 (NLRP3) inflammasome in myeloid cells.^45^ It is not clear why β-hydroxybutyrate was decreased in patients with autistic regression: hypothetical explanations include reduced production or increased ‘consumption’. A further consideration is that children with autism often have restricted eating patterns with potentially secondary poor gut health and an altered microbiome, which could be a contributor to altered butyrate production.

Given the potentially useful therapeutic effects of β-hydroxybutyrate, means of increasing this metabolite are worthy of consideration. The ketogenic diet (KD) is a high-fat and protein, and low-carbohydrate diet. β-hydroxybutyrate is a major ketone body produced as a response to the carbohydrate deprivation in KD. KD is a novel dieto-therapeutic approach with potential to reduce inflammation and modulate cell function and signalling in a variety of health disorders such as neurological disorders,^46^, ^47^, ^48^ epilepsy,^49^ cardiovascular diseases,^50^ and cancer.^51^ A growing body of research in autism has shown KD can improve social behaviour, reduce neuroinflammation and oxidative stress, and modulate the gut microbiota.^52^^,^^53^ Alternative means of butyrate production could include supplementation with butyrate salts or esters, or modification of the microbiome to increase butyrate producing microbial species.^54^^,^^55^

The second major finding was elevation of S1P, and by extension the dysregulation of sphingolipids in patients with autistic regression. S1P is a signalling lipid that acts as an important regulator in many cellular processes including inflammation, angiogenesis, cell growth, and brain and cardiac development.^56^^,^^57^ S1P serves as both an independent and dependent intracellular messenger and signals extracellularly through S1P receptors.^58^ S1P plays an important role in the innate and adaptive immune system, and modulates the host inflammatory immune system,^59^^,^^60^ and has become a key target in clinical trials for autoimmune diseases^61^^,^^62^ and other inflammatory disorders.^63^^,^^64^

Sphingolipid metabolism consists of three major metabolic pathways^65^ (*de novo* pathway, sphingomyelin and salvage pathway) which converges around the biosynthesis and catabolism of ceramide (Fig. 3c). Our work presents an interesting finding that most of the sphingolipids were elevated in autistic regression (S1P, ceramide, hexosylceramides and sulfatides). However, sphingomyelins were the only sphingolipids that were decreased in patients with autistic regression. Sphingomyelin is metabolised to ceramide by sphingomyelinase, which is activated by inflammation.^66^ Sphingomyelinase is used as a diagnostic inflammatory biomarker in neurological disorders^67^ and a potential future therapeutic target.^68^^,^^69^ The dysregulation of sphingolipid metabolism has been previously reported in an autism-specific prefrontal cortex genome-scale metabolic model, which identified S1P, ceramide and glucosylceramide as important discriminating metabolites.^70^ Therefore, S1P is an important mechanistic marker found in our autistic regression study. The modulation of S1P signalling may represent as an innovative and promising target for therapeutics.

A first limitation of our study was the focus on a subset of metabolites, and the limited CSF sample volumes available to measure and validate the compounds identified in the untargeted metabolomics studies. The second limitation is the absence of blood or urine samples to conduct simultaneous analysis with CSF to determine if a less invasive biological matrix (e.g., blood), can aid in the diagnosis of autistic regression. We detected several drug metabolites in our patients, which were generally increased in the patients with autistic regression. Most of these drugs were not reported by families in the clinical assessment, although some of the families were taking herbal or other supplements which could have contained the drug metabolites detected. It should be acknowledged that the drugs could, in theory, influence metabolic findings, and this limitation is common in the investigation of complex human disease where families seek therapeutic solutions due to the failure of conventional medicine. A further limitation was the fact that not all patients had comprehensive genetic testing, such as whole exome or genome sequencing-it is conceivable that some patients harbour genetic variants that were undetected due to incomplete testing. It is also conceivable that these twenty-two patients constitute an ‘atypical’ group of patients with autistic regression, due to the treating clinicians feeling a CSF was warranted based on the history. Future research would employ regression-specific measures to collect data in a systematic and prospective manner. Another limitation of the study is that our control group is not ‘normal’, and most controls had neurological diseases (but not autistic regression). Future studies would include comparisons with ‘healthy’ controls and an autism group without regression. Finally, although our biomarkers have shown statistical significance in the setting of this cohort study, these biomarkers are unlikely to be adequately discriminating at an individual level.

Metabolomics and lipidomics are valuable tools to profile metabolic patterns, enhancing our understanding of cellular function, immunity and biomolecular alterations associated with the onset and progression of autism. There is a scarce literature investigating molecular profiles to help define autism subgroups.^71^^,^^72^ Future research focusing on subgroups of patients with autism will be powerful to uncover the underlying biology of complex autism traits and could lead to targeted approaches in personalised medicine and new methods for pre-symptomatic risk stratification during newborn screening. Moreover, the lipidome holds promise as biomarkers of environmental influences such as unhealthy diet patterns and sleep disturbances in patients with autism.^73^ Further research understanding the complex interplay between environmental factors with neurodevelopment in autism is important for long-term wellbeing and quality of life of patients.

### Conclusion

In conclusion, CSF metabolomics is a powerful approach that provides insights into disease mechanisms. We provide discovery and validation data to show that reduced β-hydroxybutyrate and dysregulated sphingolipids are associated with autistic regression, and these metabolites offer potential as future therapeutic targets.

## Contributors

JY and RCD conceptualised and designed the experiments. VXH, HFJ, SP, RCD phenotyped patients from the electronic medical record. RW, KK, MPM, JA, TK, MC, RCD recruited study patients. JY, TAC, BJ, CL performed the experiments and data acquisition. JY, TAC, BJ, CL, RCD analysed and interpreted the data. JY, JL, SB, RCD performed validation. JY and RCD wrote the original manuscript. All authors critically revised and edited the manuscript. All authors approved the final version of the article.

## Data sharing statement

All data are provided with this paper. The deidentified datasets used and/or analysed during the current study are available from the corresponding author on reasonable request. Raw data can be accessed by researchers who provide a written study proposal, for approval by the Kids Neuroscience Centre.

## Declaration of interests

The authors have declared that no conflict of interest exists.
