# Supplementary material for: Cerebrospinal fluid metabolomics in autistic regression reveals dysregulation of sphingolipids and decreased β-hydroxybutyrate
Source: eBioMedicine. 2025 Mar 25;114:105664. doi: 10.1016/j.ebiom.2025.105664 (PMC11986237; doi:10.1016/j.ebiom.2025.105664)
Supplement: Supplemental Tables S1–S3 [file mmc1.docx]

# Supplemental Table 1: Catalogue number for chemicals and reagents.

| **Chemical/ Reagent** | **Product Number** | **Manufacturer** |
| --- | --- | --- |
| HPLC grade acetonitrile | 34851 | Sigma Aldrich (Sydney, Australia) |
| HPLC grade methanol | 34860 |  |
| Ammonium formate | 70221 |  |
| tert-Butyl methyl ether | 34875 |  |
| D_3_-tryptophan | T947202 | Toronto Research Chemicals (Toronto, Canada) |
| D_4_-kynurenine | K661003 |  |
| 13C6-arginine | A769507 |  |
| d17:1 Sphingosine | 10007902 | Sapphire Bioscience (Sydney, Australia) |
| d17:1 Sphingosine-1-Phosphate | 22498 |  |
| d18:1/17:0 Sphingomyelin | 25592 |  |
| d18:1/17:0 Ceramide | 22532 |  |
| Formic acid | T27563 | Fisher Chemical (Fair Lawn, New Jersey) |

# Supplemental Table 2: Summary of upregulated drug metabolites in untargeted metabolomics case-control studies for children with autistic regression (n=22) compared to controls (n=22) using at a p-value cut-off of 0.01, fold change and false discovery rate at 5% with *p*_FDR_ adjusted values < 0.05 as statistically significant. ↑ represents elevation and ↓ represents decreased levels.

| **Case-control Study 1 Drug Metabolite** | **Change** | **Fold Change** | **effect size** | **p_FDR_** |
| --- | --- | --- | --- | --- |
| Buspirone | **↑** | 4.90 | 1.64 | 0.004 |
| Cyclazocine | **↑** | 4.49 | 1.52 | 0.002 |
| Metoprolol | **↑** | 3.89 | 1.06 | 0.006 |
| Memantine | **↑** | 3.45 | 1.99 | 0.002 |
| Isophorone | **↑** | 3.26 | 1.84 | 0.008 |
| Carisoprodol | **↑** | 2.36 | 0.87 | 0.05 |
| Fluprednisolone | **↑** | 2.26 | 0.99 | 0.02 |
| Atagabalin | **↑** | 2.11 | 1.39 | 0.02 |
| Pramiracetam | **↑** | 2.09 | 0.34 | 0.04 |
| Succinylcholine | **↑** | 1.97 | 1.25 | 0.03 |
| Azelaic acid | **↑** | 1.75 | 1.20 | 0.02 |
| Cerulenin | **↑** | 1.64 | 1.86 | 0.01 |
| **Case-control Study 2 Drug Metabolite** | **Change** | **Fold Change** | **effect size** | **p_FDR_** |
| Rivastigmine | **↑** | 2.96 | 0.90 | 0.04 |
| Imagabalin | **↑** | 2.95 | 0.91 | 0.05 |
| Pentobarbital | **↑** | 1.93 | 0.89 | 0.04 |
| Pramiracetam | **↑** | 1.77 | 0.97 | 0.05 |
| Theophylline | **↑** | 1.72 | 0.86 | 0.05 |
| Rasagiline | **↑** | 1.61 | 1.05 | 0.04 |
| Zonisamide | **↑** | 1.50 | 1.31 | 0.05 |
| Zalcitabine | **↓** | 0.42 | 0.71 | 0.02 |

# Supplemental Table 3: Summary of statistical comparisons using non-parametric (Mann–Whitney U test) and Benjamini-Hochberg false discovery rate correction, and direction of change (of autistic regression comparted to controls) of sphingolipids in autistic regression (n=14) compared to neurodevelopmental disorder control group (n=16). In addition, autistic regression (n=14) group is compared with the other neurological control group (n=34) is presented.

| **Sphingolipid** | **Autistic regression (n=14) v neurodevelopmental disorders (n=16)** | | | **Autistic regression (n=14) v other neurological controls (n=34)** | | |
| --- | --- | --- | --- | --- | --- | --- |
|  | **Original p value** | **BH adjusted p value** | **Change Direction** | **Original p value** | **BH adjusted p value** | **Change Direction** |
| Cer (d18:1/16:0) | 0.001 | 0.008 | ↑ | 0.0001 | 0.0003 | ↑ |
| Cer (d18:1/16:1) | 0.007 | 0.02 | ↑ | 0.2 | 0.3 |  |
| Cer (d18:1/18:0) | 0.006 | 0.02 | ↑ | 0.005 | 0.009 | ↑ |
| Cer (d18:1/20:0) | 0.002 | 0.009 | ↑ | 0.0001 | 0.0003 | ↑ |
| Cer (d18:1/20:1) | 0.002 | 0.009 | ↑ | 0.0001 | 0.0003 | ↑ |
| Cer (d18:1/22:0) | 0.0002 | 0.002 | ↑ | 0.0001 | 0.0003 | ↑ |
| Cer (d18:1/24:0) | 0.0001 | 0.001 | ↑ | 0.0001 | 0.0003 | ↑ |
| Cer (d18:1/24:1) | 0.0001 | 0.001 | ↑ | 0.0001 | 0.0003 | ↑ |
| HexCer (d18:1/16:0) | 0.009 | 0.02 | ↑ | 0.0002 | 0.0006 | ↑ |
| HexCer (d18:1/18:0) | 0.01 | 0.03 | ↑ | 0.0001 | 0.0003 | ↑ |
| HexCer (d18:1/24:1) | 0.002 | 0.009 | ↑ | 0.0001 | 0.0003 | ↑ |
| HexCer (d18:2/18:0) | 0.08 | 0.2 |  | 0.005 | 0.009 | ↑ |
| HexCer (d18:2/20:0) | 0.2 | 0.3 |  | 0.1 | 0.1 |  |
| HexCer (d18:2/22:0) | 0.003 | 0.009 | ↑ | 0.003 | 0.005 | ↑ |
| HexCer (d18:2/24:1) | 0.02 | 0.04 | ↑ | 0.002 | 0.004 | ↑ |
| SM (d18:0/16:0) | 0.1 | 0.2 |  | 0.002 | 0.004 | ↓ |
| SM (d18:0/18:0) | 0.9 | 0.9 |  | 0.3 | 0.3 |  |
| SM (d18:0/20:0) | 0.3 | 0.4 |  | 0.5 | 0.5 |  |
| SM (d18:0/22:0) | 0.2 | 0.3 |  | 0.6 | 0.6 |  |
| SM (d18:1/16:0) | 0.002 | 0.03 | ↓ | 0.0001 | 0.0003 | ↓ |
| SM (d18:1/16:1) | 0.3 | 0.4 |  | 0.0002 | 0.0006 | ↓ |
| SM (d18:1/18:0) | 0.003 | 0.009 | ↓ | 0.0001 | 0.0003 | ↓ |
| SM (d18:1/18:1) | 0.8 | 0.9 |  | 0.009 | 0.02 | ↓ |
| SM (d18:1/20:0) | 0.1 | 0.2 |  | 0.02 | 0.04 | ↓ |
| SM (d18:1/20:1) | 0.4 | 0.5 |  | 0.001 | 0.003 | ↓ |
| SM (d18:1/22:0) | 0.6 | 0.7 |  | 0.09 | 0.1 |  |
| SM (d18:1/22:1) | 0.2 | 0.3 |  | 0.06 | 0.09 |  |
| SM (d18:2/16:1) | 0.9 | 0.9 |  | 0.02 | 0.03 | ↑ |
| SM (d18:2/20:1) | 0.2 | 0.3 |  | 0.1 | 0.1 |  |
| SM (d18:2/22:1) | 0.9 | 0.9 |  | 0.4 | 0.4 |  |
| SM (d18:2/24:1) | 0.9 | 0.9 |  | 0.7 | 0.7 |  |
| ST (d18:1/18:0) | 0.0001 | 0.001 | ↑ | 0.0001 | 0.0003 | ↑ |
| ST (d18:1/22:1)/LacCer (d18:1/16:0) | 0.9 | 0.9 |  | 0.6 | 0.6 |  |
| ST (d18:1/24:1)/LacCer (d18:1/18:0) | 0.5 | 0.6 |  | 0.3 | 0.3 |  |
| ST (d18:2/22:0) | 0.01 | 0.03 | ↑ | 0.003 | 0.005 | ↑ |
| ST (d18:2/24:0) | 0.0009 | 0.007 | ↑ | 0.0007 | 0.002 | ↑ |
| ST (d18:2/24:1)/LacCer (d18:2/18:0) | 0.01 | 0.03 | ↑ | 0.0001 | 0.0003 | ↑ |
| S1P 18:1 | 0.01 | 0.02 | ↑ | 0.02 | 0.03 | ↑ |
| Sph 18:0 | 0.01 | 0.03 | ↑ | 0.05 | 0.07 |  |
| Sph 18:1 | 0.2 | 0.3 |  | 0.2 | 0.3 |  |
